# Restriction spectrum imaging and diffusion kurtosis imaging for evaluating proliferation status in cervical cancer

**DOI:** 10.3389/fmed.2026.1855242

**Published:** 2026-08-06

**Authors:** Wenling Liu, Huijia Yin, Beiran Wang, Zhong Li, Wangyi Liu, Meng Zhang, Jipeng Ren, Ruifang Yan, Dongming Han

**Affiliations:** Department of MR, The First Affiliated Hospital, Henan Medical University, Weihui, China

**Keywords:** cervical cancer, diffusion kurtosis imaging, diffusion-weighted imaging, proliferation status, restriction spectrum imaging

## Abstract

**Objectives:**

To explore the diagnostic efficacy of three-compartment restriction spectrum imaging (RSI) and diffusion kurtosis imaging (DKI) in predicting proliferative status in patients with cervical cancer (CC).

**Methods:**

A total of 100 patients diagnosed with CC were enrolled in the present study. All patients were stratified into the high- (Ki-67 > 50%, *n* = 58) and low-proliferation group (Ki-67 ≤ 50%, *n* = 42). Quantitative parameters derived from RSI (*f*_1_, *f*_2_, *f*_3_), DKI (MD, MK), and conventional diffusion-weighted imaging (DWI, ADC) were compared between the two groups. Logistic regression (LR) analysis was performed to screen independent predictors and construct an integrated diagnostic model. The area under the receiver operating characteristic curve (AUC) was calculated to quantify the diagnostic performance, and the DeLong test was adopted for pairwise comparisons of AUC values.

**Results:**

Compared with patients in the low-proliferation group, those in the high- proliferation group presented significantly lower *f*_1_, MD, and ADC values, along with markedly elevated MK, *f*_2_, and *f*_3_ values (all *P* < 0.05). The corresponding AUC values were 0.84, 0.76, 0.76, 0.87, 0.79, and 0.80, respectively. LR analysis identified *f*_2_, MD, MK, and ADC as independent predictors. The integrated diagnostic model based on these four independent predictors yielded optimal diagnostic performance, with an AUC of 0.94 (95% CI: 0.87–0.98). DeLong analysis revealed that this integrated model significantly outperformed all single imaging parameters (*f*_1_, *f*_2_, *f*_3_, MD, MK, ADC), with *Z* values of 1.99, 3.11, 2.58, 4.09, 2.46, and 4.05 (all *P* < 0.05). Internal bootstrap validation confirmed the excellent stability of the combined model, with a corrected AUC of 0.92 (95% CI: 0.90–0.93).

**Conclusion:**

RSI, DKI and conventional DWI are all reliable imaging techniques for evaluating proliferative status in CC. The combined application of *f*_2_, MD, MK, and ADC parameters serves as a promising non-invasive biomarker strategy for guiding individualized treatment decision-making and prognostic stratification in patients with CC.

## Introduction

Cervical carcinoma (CC) ranks as the fourth most prevalent malignancy and constitutes a significant cause of cancer-related mortality among women worldwide. Epidemiological data indicate a persistent annual increase in CC incidence in developing countries and regions, accompanied by a rising trend of early-onset cases among young female populations (1, 2). Ki-67, a key nuclear protein essential for maintaining chromatin stability during cell division, serves as a critical biomarker for assessing abnormal proliferation and the malignant invasive potential of tumor cells (3). Numerous studies have established a strong association between elevated Ki-67 expression and rapid tumor progression, as well as poor prognostic outcomes in CC patients (4, 5). Currently, pathological biopsy is considered the gold standard for evaluating tumor proliferative activity. Nevertheless, this invasive diagnostic method is subject to inherent limitations; sampling heterogeneity and technical variations in procedure often result in detection variability, thereby compromising the accuracy and reproducibility of diagnostic outcomes (6, 7). Accordingly, the exploration of a non-invasive, accurate approach to evaluate Ki-67 expression in CC carries important clinical implications for the individualized clinical management of CC patients.

Diffusion-weighted imaging (DWI) is a robust and widely acknowledged magnetic resonance imaging (MRI) sequence, with its derived apparent diffusion coefficient (ADC) providing a quantitative measure of the diffusion characteristics of water molecules in living tissues (8). As a classical diffusion-based quantitative biomarker, ADC has been extensively employed to assess proliferative activity in various malignant tumors, including CC and Soft tissue sarcoma (9, 10). However, conventional DWI is limited by a fundamental theoretical constraint: it presupposes that water diffusion follows a homogeneous Gaussian distribution. This oversimplified model inadequately captures the complex microstructural heterogeneity of tumor parenchyma, leading to suboptimal diagnostic accuracy for the stratification of CC proliferation and indicating significant potential for technical advancement. Diffusion kurtosis imaging (DKI) represents a sophisticated advancement of traditional diffusion-weighted imaging (DWI). Unlike standard DWI, which relies on the Gaussian diffusion assumption, DKI is based on the theory of non-Gaussian water diffusion distribution. By integrating a fourth-order three-dimensional tensor into the conventional DWI framework, DKI effectively captures the intricate diffusion patterns of water molecules in biological tissues, thereby significantly enhancing the quantitative accuracy in evaluating tissue diffusion characteristics (11). Currently, research has employed DKI to assess cell proliferation status in diseases such as gastric cancer and glioma, yielding promising results (12, 13). Restriction spectrum imaging (RSI) is an innovative and advanced diffusion MRI technique. By fitting diffusion-weighted signals to a linear combination of various diffusion models, RSI facilitates precise and robust classification of *in vivo* water diffusion behaviors into three distinct tissue microstructural compartments: restricted diffusion, hindered diffusion, and free water diffusion (14). Andreassen et al. have substantiated the utility of RSI in evaluating the response to neoadjuvant therapy in breast cancer (15), while Rojo et al. have confirmed its feasibility in the differential diagnosis of prostate carcinoma (16). However, research on the application of RSI in CC is still limited. To the best of our knowledge, only Pan et al. have reported on the utility of RSI for assessing lymph node metastasis in patients with CC (17). Notably, no head-to-head comparative study of RSI and DKI for assessing tumor proliferative activity in CC has been published, and integrated diagnostic models combining parameters from multiple diffusion imaging sequences are still lacking.

Therefore, this study aims to explore the value of RSI and DKI-related quantitative parameters in differentiating the high-proliferative and low-proliferative subgroups of CC; with the expectation of developing new imaging biomarkers to achieve non-invasive detection of Ki-67 expression levels in CC.

## Materials and methods

### Study population

**Figure 1:**
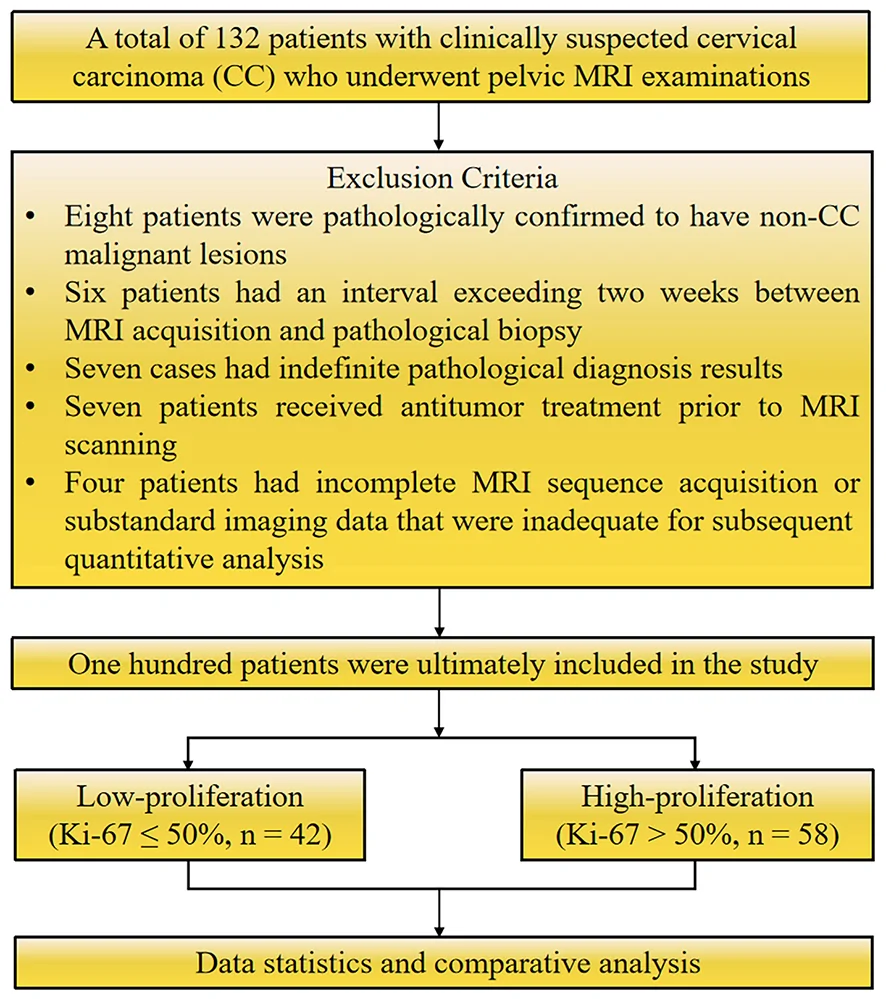
Study flowchart.

This study received approval from the institutional ethical review committee, and written informed consent was obtained from all participants prior to their enrollment. A total of 132 patients with clinically suspected CC who underwent pelvic MRI examinations were recruited between May 2021 and October 2025 following a comprehensive clinical evaluation. The specific exclusion criteria were as follows: (1) eight patients were pathologically confirmed to have non-CC malignant lesions; (2) six patients had an interval exceeding 2 weeks between MRI acquisition and pathological biopsy; (3) seven cases had indefinite pathological diagnosis results; (4) seven patients received antitumor treatment prior to MRI scanning; and (5) four patients had incomplete MRI sequence acquisition or substandard imaging data that were inadequate for subsequent quantitative analysis. Consequently, a final cohort of 100 patients was included for further analysis (Figure 1). Baseline clinical and pathological data for all enrolled patients, including age, maximum tumor diameter, International Federation of Gynecology and Obstetrics (FIGO) stage, and histological subtype, were documented for subsequent statistical analysis.

### MRI acquisition

All MRI acquisitions were conducted using a 3.0-Tesla Ingenia Elition X MR scanner (Philips Healthcare, the Netherlands), equipped with a 16-channel torso phased-array coil. Participants were positioned supine with a feet-first orientation within the magnet bore. Initial localization of cervical lesions was achieved through axial T2-weighted imaging (T2WI), employing the following acquisition parameters: repetition time/echo time (TR/TE) of 3,750/80 ms, slice thickness of 6 millimeters, interslice gap of 1 millimeter, number of excitations (NEX) set at 2, field of view (FOV) measuring 286 × 320 millimeters, and an acquisition matrix of 320 × 320. The T2WI scan duration was 1 min and 23 s. Subsequently, lesion-matched slices were acquired using the multi-b-value diffusion sequence, guided by anatomical landmarks identified in the T2WI. The slice thickness and interslice spacing were consistent with those used in the T2WI. The parameters were specifically configured as follows: TR/TE of 5,450/87 ms, b-values ranging from 0 to 3,000 s/mm^2^ (0, 25, 50, 100, 150, 200, 400, 600, 800, 1,000, 1,500, 2,000, 3,000 s/mm^2^), NEX values of 1, 1, 1, 1, 1, 1, 2, 2, 4, 6, 6, 6, 6, FOV of 400 × 300 millimeters, and a matrix of 132 × 99. Total acquisition time for the full RSI sequence reached 6 min 8 s.

### MRI data processing

All post-processing of imaging data was conducted using MATLAB R2018b (MathWorks Inc., Natick, MA, USA). Initially, the multi-b-value diffusion data underwent preprocessing to address common imaging artifacts, such as B_0_ inhomogeneity distortion, gradient nonlinearity, and eddy current artifacts. Subsequent to preprocessing, parametric pseudo-color maps for DWI, DKI, and RSI were independently generated in accordance with Equations 1, 2, 3, respectively.


$$\begin{array}{l} S_{b}/S_{0}=\text{exp}\left(-b\times \text{ADC}\right) \end{array}$$ (1)

For the DWI model (Equation 1), ADC represents the apparent diffusion coefficient; b denotes the diffusion-sensitizing gradient factor; and *S*_*a*_/*S*_*b*_ correspond to the tissue signal amplitudes at b-values of 0 s/mm^2^ and 800 s/mm^2^, respectively (18).


$$\begin{array}{l} S_{b}=S_{0}\times \text{exp}\left(-b\times D_{\text{app}}+b^{\text{2}}\times {D_{\text{app}}}^{\text{2}}\times K_{\text{app}}/\text{6}\right) \end{array}$$ (2)

For the DKI model (Equation 2), *D*_app_ refers to the apparent diffusivity and *K*_app_ represents the apparent kurtosis. The mean *D*_app_ (MD) is defined as the average diffusion coefficient corrected for non-Gaussian diffusion bias, whereas the mean *K*_app_ (MK) quantifies the average deviation of water diffusion from a perfect Gaussian distribution (19).


$$\begin{array}{l} S\left(b\right)=f_{1}{e^{-bD}}^{\text{1}}+f_{2}{e^{-bD}}^{\text{2}}+f_{3}{e^{-bD}}^{\text{3}},D\text{1}<D\text{2}<D\text{3} \end{array}$$ (3)

For the RSI model (Equation 3), the selected b-value gradient set encompasses low (0–200 s/mm^2^), intermediate (200–1,000 s/mm^2^), and high (1,000–3,000 s/mm^2^) diffusion weighting ranges. This selection facilitates the effective differentiation of intracellular restricted water, extracellular hindered water, and interstitial free water within tumor tissues. To mitigate inter-subject signal variability and systematic bias, all voxel-wise signal intensities were normalized to the maximum signal intensity across all b-values. Voxel-level RSI parameter fitting was conducted using a constrained nonlinear least-squares Levenberg–Marquardt algorithm. The volume fractions of all compartments were rigorously constrained within the physiological range of 0 to 1. Voxels exhibiting invalid signals or unsuccessful fitting results were excluded from further analysis. The parameters *f*_1_, *f*_2_, and *f*_3_ denote the volume fractions of restricted diffusion, hindered diffusion, and free water compartments, while *D*1, *D*2, and *D*3 represent the ADCs of these three distinct microstructural compartments. To mitigate model overfitting, ensure the linearization of the RSI algorithm, and maintain comparability of compartment-specific volume fractions, the values of *D*1, *D*2, and *D*3 were constrained to theoretical standardized values of 0.5 × 10^−3^ mm^2^/s, 1.3 × 10^−3^ mm^2^/s, and 3.0 × 10^−3^mm^2^/s, respectively. This approach is supported by existing theoretical and experimental evidence (20). Within the context of the three-compartment Gaussian diffusion model, the diffusion coefficients for the three types of tissue water exhibit relatively stable physiological ranges in cervical cancer. Fixing these ADCs is a standard methodological practice within this RSI framework, as it reduces the number of free parameters during nonlinear fitting, minimizes the risk of overfitting, and enhances fitting stability, particularly for images obtained at high b-values with reduced signal-to-noise ratio (SNR). Furthermore, the use of standardized fixed diffusion coefficients improves the reproducibility of RSI-derived volume fractions across different research cohorts and MRI devices.

### Parameter computation

Two radiologists with rich diagnostic experience performed manual segmentation of the entire tumor volume using ITK-SNAP software (Version 3.8.0; http://www.itksnap.org). Both readers were fully masked to all histopathological results and clinical baseline data during the procedure. Regions of interest (ROIs) were outlined along the tumor periphery on DWI images. To exclude regions presenting obvious cystic degeneration, necrosis, hemorrhage and calcification, ROIs were cross-checked against the matched T2WI sequences. After finishing ROI segmentation on DWI, these whole-tumor ROIs were automatically propagated to the aforementioned parametric maps, including ADC, MD, MK, *f*_1_, *f*_2_ and *f*_3_. Quantitative values of each imaging parameter were then extracted for subsequent statistical analysis.

### Ki-67 analysis

Immunohistochemical analysis of Ki-67, a pivotal biomarker for assessing tumor proliferative activity, was performed utilizing murine anti-human monoclonal antibodies (clone MIB-1, DAKO, Denmark) to facilitate quantitative evaluation. For each pathological section, five high-power fields (×400 magnification) exhibiting the highest density of Ki-67-positive tumor cells, referred to as hotspots, were selected for analysis. In each hotspot, a systematic quantification of 100 tumor cells was conducted to determine the Ki-67 nuclear positive expression rate. Using a Ki-67 labeling index of 50% as the predefined cutoff value, specimens with a Ki-67 positivity rate ≤ 50% were categorized into the low-proliferation group, whereas specimens with a Ki-67 positivity rate > 50% were classified into the high-proliferation group. This 50% cutoff threshold has been extensively validated and widely adopted in previous cervical cancer proliferation studies as a clinically meaningful stratification criterion for evaluating tumor malignant potential (9).

### Data analysis

Intraclass correlation coefficients (ICCs) were utilized to assess inter-reader agreement for quantitative metrics derived from DWI, DKI, and RSI. An ICC value exceeding 0.75 was deemed indicative of excellent inter-reader reproducibility (21). Prior to comparing imaging parameters between the low- and high-proliferation groups, statistical tests were selected based on the distribution patterns of the data. Specifically, the Mann–Whitney *U*-test was employed for data not conforming to a normal distribution, the independent-samples *t*-test was applied for normally distributed data, and the chi-square test was used for categorical variables. Binary logistic regression (LR) analysis (Enter method) was conducted to identify independent predictive indicators and to construct an integrated diagnostic model. The variance inflation factor (VIF) is used to assess multicollinearity; a value < 10 is considered to indicate the absence of significant multicollinearity. Internal validation of the model was performed using bootstrap resampling with 1,000 iterations. The area under the receiver operating characteristic curve (AUC) was calculated to evaluate diagnostic performance, and the DeLong test was employed for pairwise comparisons of AUC values. All statistical analyses were executed using MedCalc software (Version 15.0; MedCalc Software, Belgium), with statistical significance defined as a two-sided *P* value of < 0.05.

## Results

### Baseline data

The final study cohort comprised 100 patients with CC, who were dichotomized into two subgroups based on Ki-67 proliferative status: 58 patients in the high-proliferation group and 42 in the low-proliferation group. Baseline clinical and pathological comparison showed that the low-proliferation subgroup exhibited a higher proportion of adenocarcinoma. No significant intergroup differences were detected in age, maximum tumor diameter, and FIGO stage between the two cohorts. The detailed results are shown in Table 1.

**Table 1:** Comparison of different variables between high- and low-proliferation groups.

Variables	High-proliferation (*n* = 58)	Low-proliferation (*n* = 42)	*t*/χ^2/^*Z* value	*P*-value
Age (years)	53.55 ± 13.08	54.05 ± 9.08	−0.21	0.833^a^
Maximum diameter (mm)	35.30 (20.88, 51.15)	27.90 (18.43, 44.83)	−1.27	0.204^b^
FIGO Stage (%)			0.54	0.461^c^
I–II	48/58 (82.76%)	37/42 (88.10%)		
III–IV	10/58 (17.24%)	5/42 (11.90%)		
Subtype (%)			4.34	0.037^c^
Squamous cell carcinoma	52/58 (89.66%)	31/42 (73.81%)		
Adenocarcinoma	6/58 (10.34%)	11/42 (26.19%)		
Parameters				
*f*_1_	0.51 ± 0.10	0.66 ± 0.11	−6.71	< 0.001^a^
*f*_2_	0.12 (0.10, 0.14)	0.07 (0.05, 0.10)	−4.98	< 0.001^b^
*f*_3_	0.39 ± 0.11	0.25 ± 0.12	5.64	< 0.001^a^
MD (×10^−3^mm^2^/s)	1.65 (1.50, 1.76)	1.89 (1.67, 2.24)	−4.39	< 0.001^b^
MK	0.73 (0.65, 0.83)	0.48 (0.39, 0.58)	−6.27	< 0.001^b^
ADC (×10^−3^mm^2^/s)	1.09 ± 0.20	1.31 ± 0.21	−5.12	< 0.001^a^

Letters a, b and c correspond to comparisons made by independent *t*-test, Mann–Whitney *U*-test, and chi-squared test, respectively. High-proliferation = Ki-67 > 50%, Low-proliferation = Ki-67 ≤ 50%.

### Interobserver agreement analysis

Quantitative assessment of inter-reader reproducibility demonstrated that the ICCs for all included imaging parameters—encompassing DWI-derived ADC, DKI-based MD and MK, as well as RSI-related *f*_1_, *f*_2_, and *f*_3_–exceeded 0.75. These results confirmed excellent consistency in quantitative measurements between the two radiologists. To minimize inter-reader measurement bias and ensure the robustness of subsequent statistical analyses, the average values obtained from the two independent observers were utilized for all subsequent data analyses.

### Comparison of parameters

**Figure 2:**
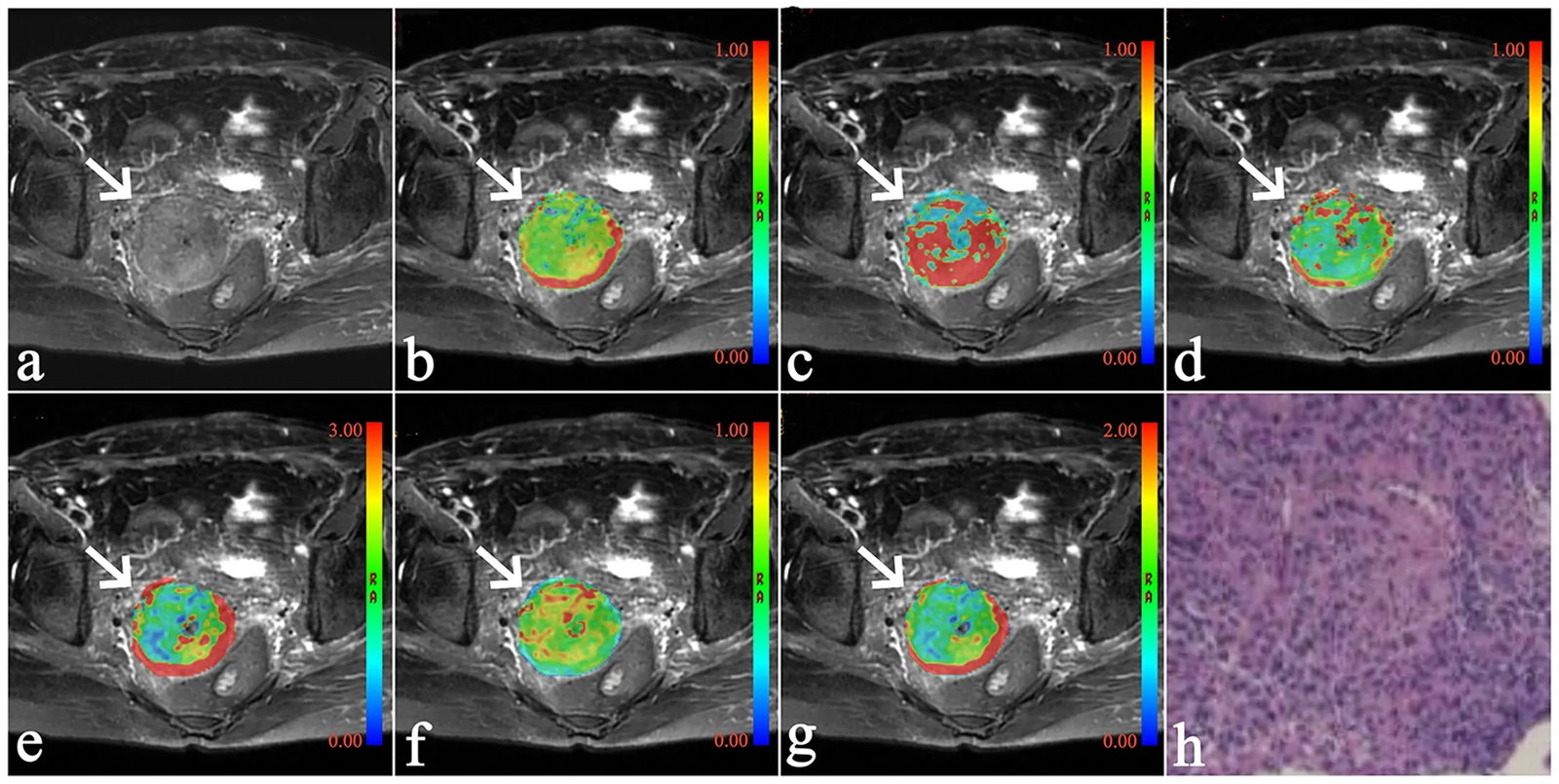
A 75-year-old woman with cervical cancer (Ki-67 = 70%). **(a)** Map of T2-weighted imaging; **(b)** Pseudo colored map of *f*_1_; **(c)** Pseudo colored map of *f*_2_; **(d)** Pseudo colored map of *f*_3_; **(e)** Pseudo colored map of MD; **(f)** Pseudo colored map of MK; **(g)** Pseudo colored map of ADC; **(h)** Pathological images (original magnification, × 100).

**Figure 3:**
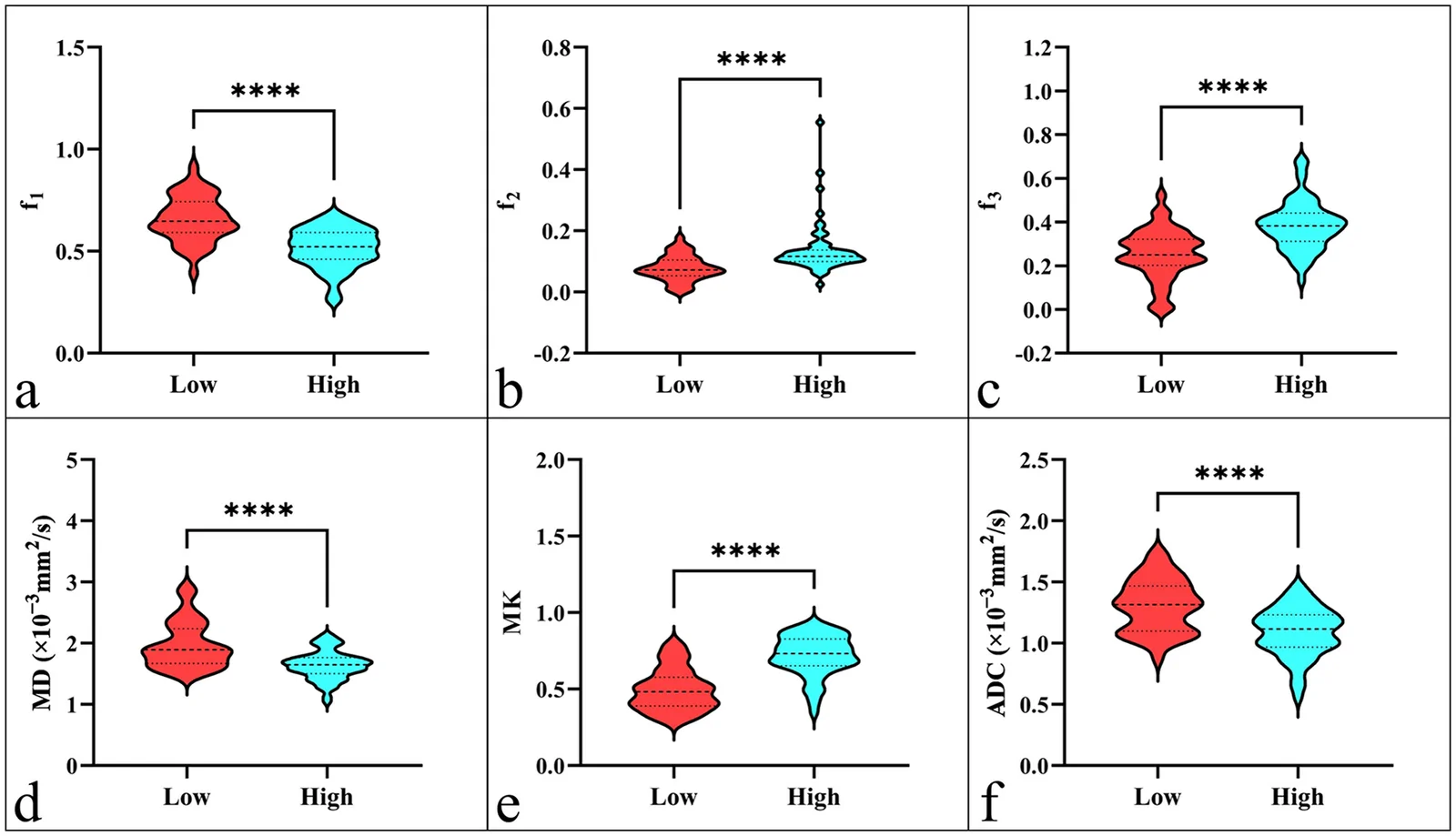
Boxplots of various parameters in high-proliferation and low-proliferation cervical cancer: **(a)** *f*_1_; **(b)** *f*_2_; **(c)** *f*_3_; **(d)** MD; **(e)** MK; and **(f)** ADC. ****P < 0.0001, and ***P < 0.001.

Compared with the low-proliferation subgroup, the high-proliferation cohort presented significantly lower values of DWI-derived ADC, DKI-derived MD, and RSI-based *f*_1_ parameters. In contrast, the high-proliferation group yielded remarkably higher DKI-derived MK, RSI-based *f*_2_, and *f*_3_ values. All the above intergroup differences were statistically significant (all *P* < 0.05), as summarized in Table 1 and Figures 2, 3.

### Independent predictors

The evaluated indicators covered demographic and clinical profiles (age, maximum tumor diameter, histological subtype and FIGO stage), as well as quantitative parameters derived from DWI, DKI and RSI (ADC, MD, MK, *f*_1_, *f*_2_, and *f*_3_). Univariate regression analysis firstly identified histological subtype, *f*_1_, *f*_2_, *f*_3_, MD, MK and ADC as statistically significant predictors (all *P* < 0.05). All above significant variables were subsequently entered into the multivariate regression model. The multivariate analysis further confirmed that *f*_2_, MD, MK and ADC were independent predictors for tumor proliferative status, with respective *P* values of 0.036, 0.045, 0.026 and 0.026 (Table 2).

**Table 2:** Univariate and multivariate LR analyses.

Variables	Univariate analyses			Multivariate analyses	
	OR^*^(95% CI)	*P*-value	VIF	OR^*^(95% CI)	*P*-value
Age (years)	0.96 (0.64 ~ 1.43)	0.831	1.078	/	**/**
Maximum diameter (mm)	1.24 (0.82 ~ 1.87)	0.305	1.429	/	**/**
FIGO Stage (%)	1.17 (0.77 ~ 1.77)	0.463	1.447	/	**/**
Subtype (%)	1.53 (1.01 ~ 2.31)	0.043	1.103	2.38 (0.91 ~ 6.24)	0.077
*f*_1_	0.16 (0.07 ~ 0.35)	< 0.001	5.524	1.94 (0.01 ~ 343.94)	0.802
*f*_2_	8.52 (3.04 ~ 23.91)	< 0.001	2.396	111.55 (1.36 ~ 9,152.71)	0.036
*f*_3_	4.30 (2.21 ~ 8.38)	< 0.001	5.061	116.50 (0.36 ~ 37,602.30)	0.106
MD (×10^−3^mm^2^/s)	0.26 (0.14 ~ 0.50)	< 0.001	1.718	0.30 (0.09 ~ 0.98)	0.045
MK	6.44 (3.24 ~ 12.83)	< 0.001	1.507	6.53 (1.25 ~ 34.07)	0.026
ADC (×10^−3^mm^2^/s)	0.30 (0.17 ~ 0.53)	< 0.001	1.647	0.08 (0.01 ~ 0.74)	0.026

OR^*^ = odds ratio per 1 standard deviation increment; 95% CI = 95% confidence interval, VIF = variance inflation factor. All factors with a *P*-value < 0.05 and a VIF < 10 in univariate Logistic Regression (LR) analysis were incorporated into subsequent multivariate LR analysis.

### Diagnostic efficacy

**Figure 4:**
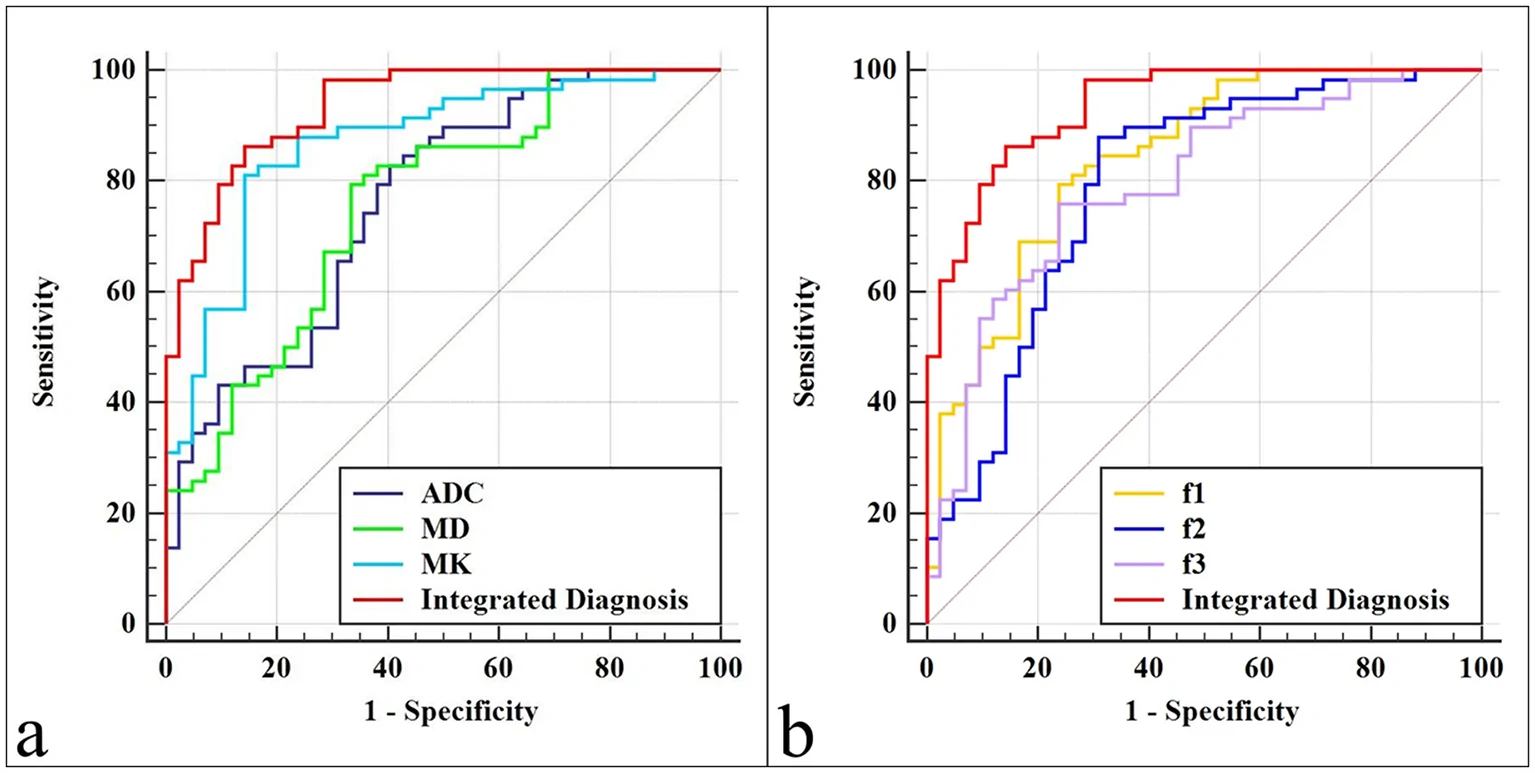
Receiver operating characteristic (ROC) curves for diagnostic performance evaluation of different quantitative parameters and the integrated diagnosis model. **(a)** ROC curves of ADC, MD, MK and the integrated diagnosis model; **(b)** ROC curves of f_1_, f_2_, f_3_ and the integrated diagnosis model. The integrated model was established based on f_2_, MD, MK, and ADC.

**Figure 5:**
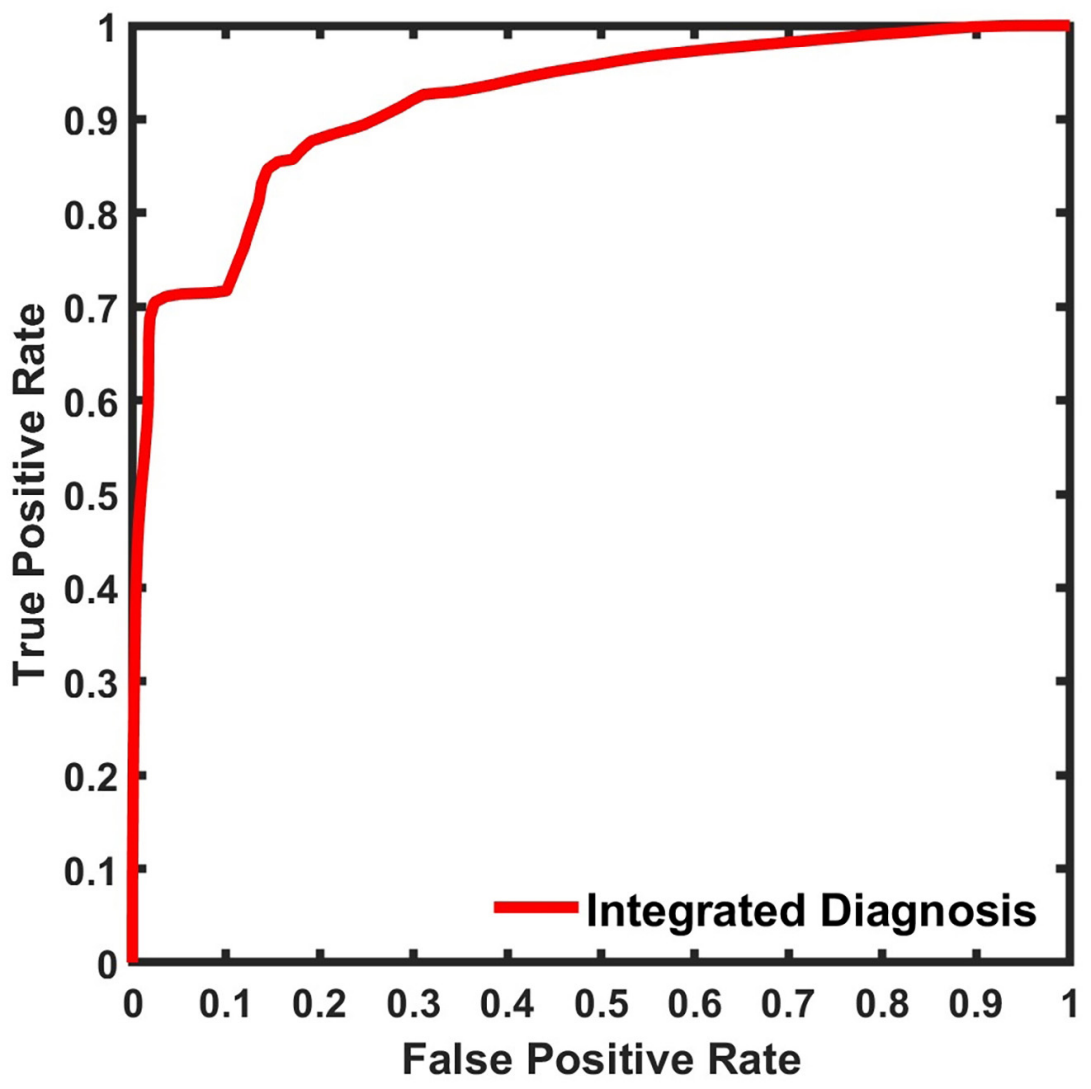
The areas under receiver-operator characteristic (ROC) curve for internal validation (bootstrap) of the integrated model (*f*_2_, MD, MK, and ADC).

The integrated predictive model consisting of four independent indicators (*f*_2_, MD, MK, and ADC) achieved favorable diagnostic efficacy, with an AUC of 0.94 (95% CI: 0.87–0.98), a sensitivity of 86.21%, and a specificity of 85.71%. Bootstrap internal validation further verified the robust stability of this combined model, with a corrected AUC value of 0.92 (95% CI: 0.90–0.93). DeLong test analysis demonstrated that the integrated model was significantly superior to all individual imaging parameters. The respective AUC values of single parameters (*f*_1_, *f*_2_, *f*_3_, MD, MK, ADC) were 0.84, 0.79, 0.80, 0.76, 0.87, and 0.76, and the corresponding *Z* values were 1.99, 3.11, 2.58, 4.09, 2.46, and 4.05 (all *P* < 0.05). Detailed results are presented in Figures 4, 5 and Table 3.

**Table 3:** ROC analysis of different variables.

Variables	AUC (95% CI)	Specificity	Sensitivity	Cutoff	Comparison with integrated diagnosis
*f*_1_	0.84 (0.75 ~ 0.91)	76.19%	79.31%	0.60	*Z* = 1.99, *P* = 0.046
*f*_2_	0.79 (0.70 ~ 0.87)	69.05%	87.93%	0.09	*Z* = 3.11, *P* = 0.002
*f*_3_	0.80 (0.70 ~ 0.87)	76.19%	75.86%	0.31	*Z* = 2.58, *P* = 0.001
MD (×10^−3^mm^2^/s)	0.76 (0.66 ~ 0.84)	66.67%	79.31%	1.78	*Z* = 4.09, *P* < 0.001
MK	0.87 (0.79 ~ 0.93)	81.03%	85.71%	0.64	*Z* = 2.46, *P* = 0.014
ADC (×10^−3^mm^2^/s)	0.76 (0.67 ~ 0.84)	59.52%	82.76%	1.27	*Z* = 4.05, *P* < 0.001
Integrated Diagnosis	0.94 (0.87 ~ 0.98)	85.71%	86.21%	0.56	/

DWI = ADC, Integrated Diagnosis = MD + MK + ADC+ *f*_2_, 95% CI = 95% confidence interval.

## Discussion

As a conventional diffusion-based MRI sequence, DWI quantifies the Brownian motion of water molecules in living tissues based on the theoretical hypothesis of homogeneous Gaussian diffusion distribution (18). As the core quantitative metric of DWI, ADC is widely recognized as a reliable surrogate index for reflecting the degree of water diffusion freedom in biological tissues. Previous Ki-67-related tumor proliferation studies have demonstrated that highly proliferative cervical lesions possess more compact cellular structure and stronger malignant aggressiveness. Such microstructural characteristics greatly restrict the diffusion movement of water molecules, thereby leading to decreased ADC values in high-proliferative CC lesions (9, 22). Consistent with the findings of previous studies, the present research also identified significantly lower ADC levels in the high-proliferation group than in the low-proliferation group. This consistent outcome further validates the capability of DWI to serve as a non-invasive imaging tool for evaluating Ki-67-based tumor proliferative activity in CC.

Unlike conventional Gaussian-based DWI, DKI characterizes *in vivo* water diffusion behavior following a non-Gaussian distribution, enabling more sophisticated evaluation of tissue microstructural heterogeneity (11). The core quantitative indicators of DKI include MD and MK. Specifically, MK reflects the overall complexity of tissue microscopic structures, whereas MD represents the average diffusion level of tissue water molecules. Previous clinical studies have validated the correlation between DKI metrics and Ki-67 proliferative activity in multiple malignancies. Li et al. confirmed the predictive potential of DKI parameters for Ki-67 status in soft-tissue sarcoma (23). Similarly, Cui et al. reported that elevated Ki-67 expression was correlated with higher kurtosis values and reduced diffusion coefficients in rectal carcinoma lesions (24). In terms of CC research, only Su et al. have previously explored the association between DKI parameters and Ki-67 expression based on a 53-case cohort (25). Their study demonstrated that highly proliferative CC lesions with compact cellular arrangement possess more intricate microstructures and severe water diffusion restriction, which corresponds to increased MK and decreased MD values (12, 23, 24). Consistent with the above evidence, our large-sample cohort of 100 CC patients yielded identical results: the high-proliferation subgroup presented significantly higher MK and lower MD values. Furthermore, both MK and MD were verified as independent imaging predictors for differentiating CC proliferative grades. These consistent findings strongly support the reliability of DKI metrics as valid non-invasive biomarkers for evaluating Ki-67-mediated tumor proliferation in CC.

Three-compartment RSI is capable of categorizing *in vivo* water diffusion behaviors in biological tissues into three independent components: restricted diffusion, hindered diffusion, and free water diffusion (26). The three core RSI parameters (*f*_1_, *f*_2_, and *f*_3_) correspond to the volume fractions of the above three diffusion compartments, and the sum of these three fractional values theoretically equals 1.0 (27). In this study, distinct intergroup differences in RSI metrics were identified. Specifically, the high-proliferation CC subgroup displayed significantly lower *f*_1_ values and higher *f*_2_ and *f*_3_ values than the low-proliferation counterpart.

Such variations are likely driven by comprehensive alterations in tumor microenvironment and microstructure induced by active cell proliferation (11, 28). Rapid division of tumor cells results in dense cellular crowding, which compresses the intracellular compartment and limits the diffusion of intracellular water, thereby decreasing the volume fraction of restricted diffusion (*f*_1_). Meanwhile, massive cell proliferation raises interstitial pressure and remodels the extracellular matrix, leading to expansion of extracellular space and a rise in hindered diffusion fraction (*f*_2_). Moreover, malignant tumors with high proliferative activity are often accompanied by active angiogenesis and disordered microcirculation, which further increases interstitial free water content and elevates the free water fraction (*f*_3_). In addition, inherent intratumoral heterogeneity of CC and the signal weighting characteristics of the multi-b-value diffusion sequence may also produce certain influences on the final RSI parameters. It should be noted that the above interpretations are inferences based on imaging manifestations and published evidence. Since this study did not perform pixel-level correlation analysis with microscopic pathology, the exact pathological and molecular mechanisms behind these parametric changes remain unclear. Further prospective studies combining *in-vivo* imaging and matched histopathological examination are required to elaborate the underlying mechanisms.

However, the results of this study do not fully align with the previous research conclusions regarding rectal cancer and lung cancer (24, 29). We speculate that two primary factors account for such interstudy discrepancies. First, different tumor types (e.g., cervical carcinoma, rectal cancer, and lung cancer) possess unique biological properties and microenvironmental characteristics, leading to inherent heterogeneity in diffusion compartment alterations across malignancies. Second, divergent RSI scanning protocols and customized *b*-value fitting strategies among different studies may directly affect the quantitative calculation of water compartment volume fractions, resulting in inconsistent parametric trends. Furthermore, considering the currently limited clinical evidence regarding RSI application in CC proliferation evaluation, further rigorous studies are still warranted.

Despite the promising clinical potential of DWI and RSI for evaluating proliferative features in CC, the present study has several inherent methodological limitations that need to be acknowledged. First, this was a single-center retrospective study with a relatively limited sample size, which inevitably restricts the external validity and generalizability of the derived conclusions. Second, the diagnostic performance of the integrated model was only evaluated via bootstrap internal validation, and no independent external cohort was introduced for further verification. The absence of external validation may limit the extrapolation of the model to multi-center clinical scenarios. Third, the RSI acquisition protocol adopted in this study was referenced from previous published studies and was not specifically optimized for the imaging characteristics of CC. The absence of CC-specific parameter optimization may compromise the optimal diagnostic performance and stability of RSI metrics in evaluating tumor proliferation. Fourth, all RSI sequences in this work were acquired based on single-shot echo-planar imaging (SS-EPI). Given the inherent drawbacks of SS-EPI, including magnetic susceptibility artifacts and limited spatial resolution, several small-volume CC lesions were excluded from the final analysis. Such exclusion was attributed to poor image visualization quality, which hindered accurate ROI segmentation and reliable quantitative parameter extraction. Fifth, stratified analysis by histological subtype (squamous cell carcinoma vs. adenocarcinoma) was not conducted in the current study. Future research should expand the sample size, enroll multi-center independent cohorts for external validation, standardize RSI acquisition and post-processing procedures, and perform stratified analyses based on clinicopathological features such as histological subtype and tumor stage. Such efforts will help obtain more stable and reproducible results, further validating the clinical applicability of RSI for non-invasive Ki-67 proliferative assessment in CC.

## Conclusion

In summary, conventional DWI, DKI, and RSI are all reliable non-invasive imaging approaches for assessing the proliferative status of CC. The integrated diagnostic model composed of *f*_2_, MD, MK, and ADC parameters yields the optimal diagnostic performance in discriminating high-proliferative from low-proliferative CC lesions, and represents a promising non-invasive biomarker strategy for clinical application. Further large-scale multicenter studies are required to validate the generalizability and translational value of this model.

## Data Availability

The raw data supporting the conclusions of this article will be made available by the authors, without undue reservation.
